# Supplementary material for: Preparing Well for Esophageal Endoscopic Detection Using a Hybrid Model and Transfer Learning
Source: Cancers (Basel). 2023 Jul 26;15(15):3783. doi: 10.3390/cancers15153783 (PMC10417640; doi:10.3390/cancers15153783)
Supplement: Supplementary file 1 [file cancers-15-03783-s001.zip › cancers-2457230-supplementary.pdf]

cancers

MDPI

*Supplementary Material*

# Preparing Well for Esophageal Endoscopic Detection Using A Hybrid Model and Transfer Learning

**Chu-Kuang Chou [1,2], Hong-Thai Nguyen [3], Yao-Kuang Wang [4,5,6], Tsung-Hsien Chen [7], I-Chen Wu [5,6], Chien-Wei Huang [8,9,*] and Hsiang-Chen Wang [3,10,*]**

[1] Division of Gastroenterology and Hepatology, Department of Internal Medicine, Ditmanson Medical Foundation Chia-Yi Christian Hospital, Chiayi 60002, Taiwan; vacinu@gmail.com (C.-K.C.)
[2] Obesity Center, Ditmanson Medical Foundation Chia-Yi Christian Hospital, Chiayi 60002, Taiwan
[3] Department of Mechanical Engineering, National Chung Cheng University, Chia-Yi 62102, Taiwan; nguyenhongthai194@gmail.com
[4] Division of Gastroenterology, Department of Internal Medicine, Kaohsiung Medical University Hospital, Kaohsiung Medical University, Kaohsiung City 80756, Taiwan; fedwang@gmail.com
[5] Department of Medicine, Faculty of Medicine, College of Medicine, Kaohsiung Medical University, Kaohsiung City 80756, Taiwan; minicawu@gmail.com
[6] Graduate Institute of Clinical Medicine, College of Medicine, Kaohsiung Medical University, Kaohsiung City 80756, Taiwan
[7] Department of Internal Medicine, Ditmanson Medical Foundation Chia-Yi Christian Hospital, Chiayi 60002, Taiwan; cych13794@gmail.com
[8] Department of Gastroenterology, Kaohsiung Armed Forces General Hospital, Kaohsiung City 80284, Taiwan
[9] Department of Nursing, Tajen University, 20, Weixin Rd., Yanpu Township, Pingtung 90741, Taiwan
[10] Hitspectra Intelligent Technology Co., Ltd., Kaohsiung City 80661, Taiwan
* Correspondence: forevershiningfy@yahoo.com.tw (C.-W.H.); hcwang@ccu.edu.tw (H.-C.W.)

## S1. Feature Extraction Module – The EfficientNet

The parameters of the model were increased. The depth of the model evolved from the original CNNs (VGG16 [26] and increased to Resnet-family [23] such as Resnet34, Resnet50, and Resnet101. However, the vanishing gradient problem was encountered when the models increased the depth dimension, but the gradient value was almost unchanged, leading to the increase of convolution layers do not contribute to the learning of the deep learning model. To overcome this problem, the residual block structure of the Resnet structure was established, which prevented increase in connections among layers and increased the efficiency of the learning model. However, increasing the weights made the parameter size of the model cumbersome, so the model was scaled.

One of the problems in designing a CNN network is model scaling. Accuracy increases with model size, but increasing the model size has not been standardized. This is an empirically based process that requires repeated experiments with different model sizes to achieve acceptable accuracy and is constrained by system resources. This method consumes considerable amounts of time and effort, and a generated model is not usually optimal.

In 2019, EfficientNet, a method for addressing the problems of model scaling, was proposed [31]. The conceptualized ideal of the EfficientNet is an attempt to improve prediction accuracy by fitting a CNN model in three dimensions: depth, width, and resolution. The depth of a CNN network corresponds to the number of layers in a network. Width refers to the number of neurons in each layer or the number of filters in each Conv layer (the number of channels of an output). Resolution is the height and width of the input image.

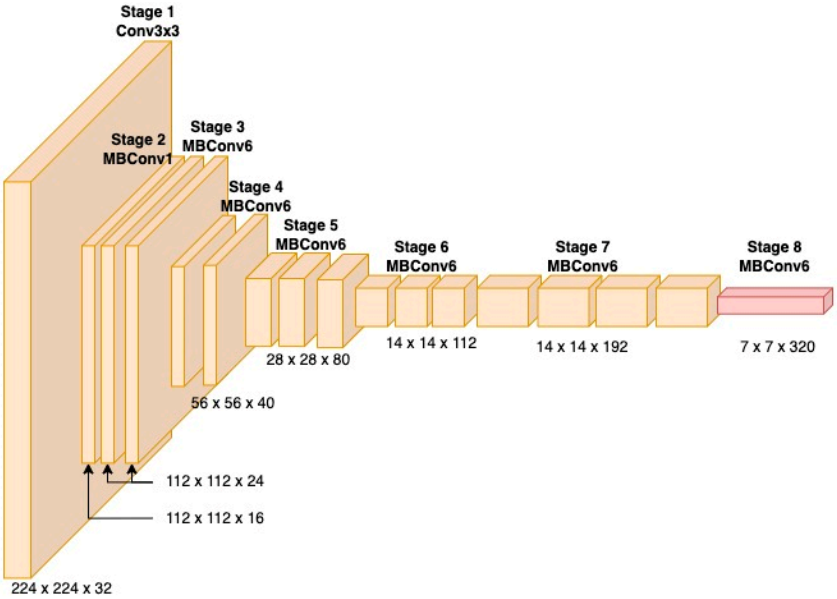


**Figure S1.** Architecture of the EfficientNet-B0 to the extracted layer (Stage 8) with feature size 7 x 7 x 320.

## S2. Vision Transformers

The Transformers structure is a permutation-equivariant architecture, which means that they produce the same amount of permutation output as the same permuted input. Typically, Transformers input is a sequence. These input feature vectors are embedded in their positions, making them have position-aware capabilities. These position embedding vectors can learn positional relationships among other embedded patches within an image. Vision Transformers is a model specifically designed for image classification.

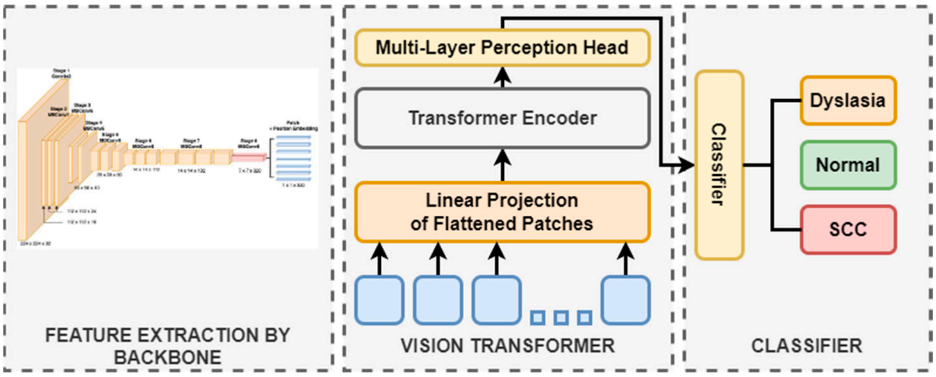


**Figure S2.** Hybrid model architecture.

## S3. System Pipeline

A platform pipeline is shown in Figure S3. It has two main parts, namely training phase and deployment phase. In the training phase, data are managed and automatically updated and downloaded during training via a cloud database, such as MongoDB and google cloud. Data are automatically downloaded during training, and the best trained-weights are automatically updated in the system. In the deployment phase, a user interface is developed to display the parameters on the screen.

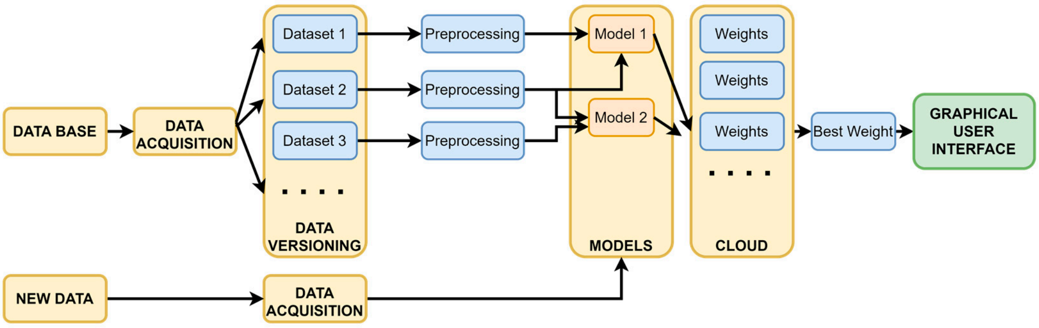


**Figure S3.** System pipeline.

## S4. Performance measures

The formulas for determining accuracy, precision, recall, and F1-score are as follows:

$$Accuracy = \frac{TN + TP}{TN + FP + TP + FN},$$

$$Precision = \frac{TP}{TP + FP},$$

$$Recall = \frac{TP}{TP + FN},$$

$$f1 - score = 2 \times \frac{Precision \times Recall}{Precision + Recall},$$

where true positive (TP) indicates that the number of positive samples is correctly classified. True negative (TN) indicates that the number of negative samples is correctly classified. False positive (FP) indicates that the number of negative samples is wrongly classified as positive. False negative (FN) indicates that the number of positive samples is wrongly classified as negative.

Receiver Operating Characteristics (ROC) depicts the correlation between two metrics evaluated through the Cartesian coordinate system in which the x-axis represents the False Positive Rate (FPR), and the y-axis represents the True Positive Rate (TPR) [S1]. The ROC curve is used to evaluate the results of a prediction based on two axes of sensitivity and characteristic. The best possible prediction method will yield a graph that is a point in the upper left corner of the ROC space. The area under the curve (AUC) indicates how good a classifier is. The random predictor will result in a line making an angle of 45 degree with the horizontal axis, from the bottom left to the top right: this is because, as the threshold increases, there will be the same number of true positives. and false positives are reduced. In other words, an AUC value of 0.5 represents a poor classification, while the AUC close to a value of 1 represents an efficient classification.

## S5. The training and validation accuracies and losses

Our proposed models achieved convergence very early from the 25th epoch, whereas classification accuracy continues to improve throughout training.

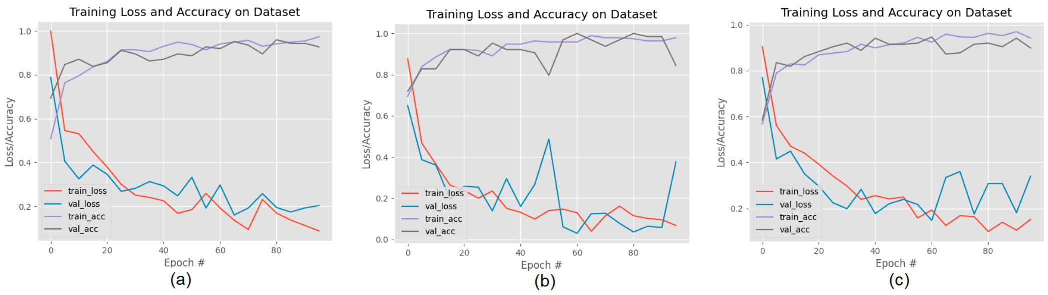

**Figure S4.** The training and validation accuracies and losses of training for (a) WLI (b) NBI, and (c) WLI + NBI dataset.

## S6. Attention matrix

The value of the attention matrix is determined by calculating the dot product.

$$Attention\ matrix\ (Q, K, V) = softmax(\frac{QK^T}{\sqrt{d_k}})V, \quad (1)$$

where input is a set of queries Q, keys K $\in R^{T \times d_k}$, and values V $\in R^{T \times d_v}$, in which T is the sequence length, and dk and dv are the hidden dimensionalities for queries/keys and values, respectively. Therefore, the attention matrix uses queries and keys to evaluate correlation with values. The product between the queries and keys shows how well each element in a sequence correlates with the other elements. This information is scaled with values. Therefore, to visualize how the attention mechanism works, we are forced to access the attention matrix, which will provide us with a 49 × 49 × 320 (or 7 × 7 × 320) image. By using an input image, we can visualize the 320 channels. Given the numerous attention heads in our network, we can survey anyone. After the position value is discarded (remaining with shape 49 = 7 × 7), information flows through different positions in the feature. This is also equivalent to what the activation weights look like across layers.

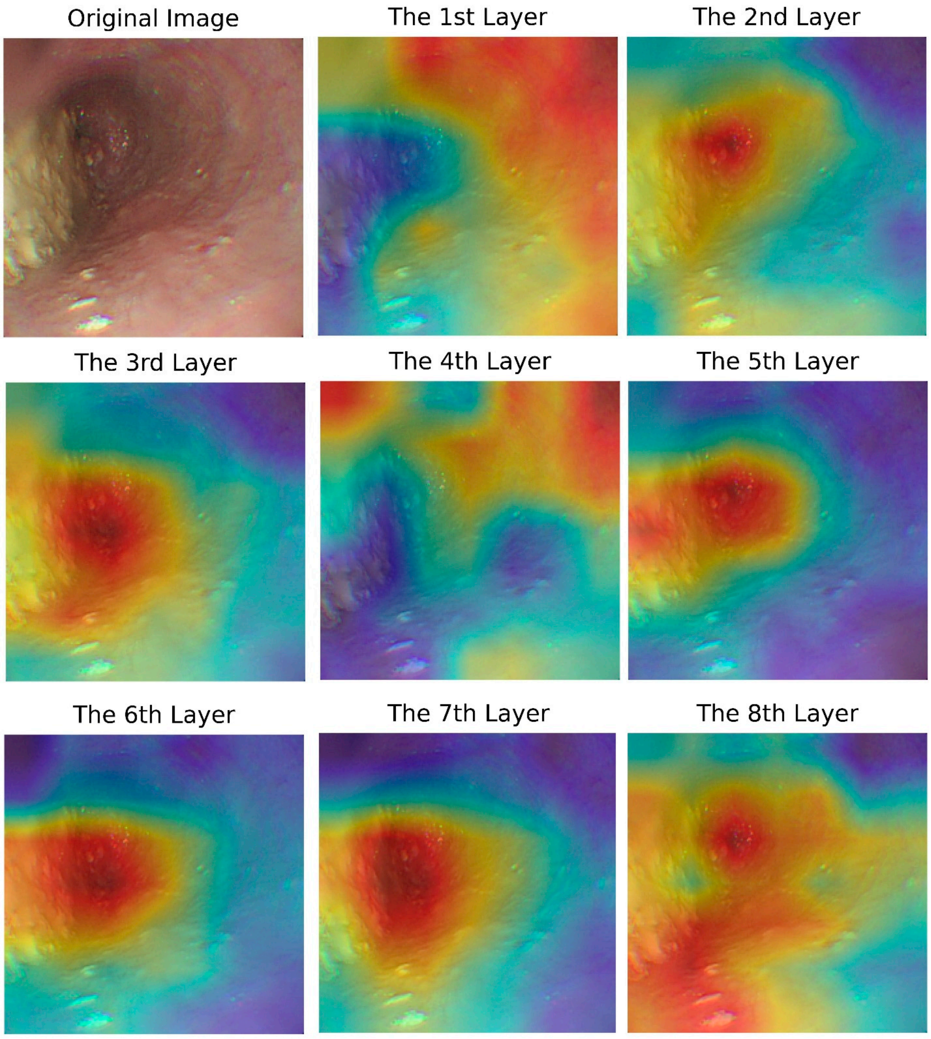


**Figure S5.** Visualization of feature maps of Attention layers. Feature maps vary from layer to layer leading to inconsistencies in detection results.

## S7. Attention Rollout

Attention Rollout: At every Transformer block, we have an attention matrix *Aij* that defines how much attention is going to flow from token *j* in the previous layer to token *i* in the next layer. The layers in the Transformers network are connected by the residual connections.

Therefore, to compute the Attention Rollout, an attention graph is constructed to represent residual connections. The values in layer $l + 1$ are determined through layers $l$ according to the following formula:

$$A = 0.5W_{att} + 0.5I, \quad \text{(S1)}$$

where $A$ is the raw attention updated by residual connections, $W_{att}$ is the attention matrix, and $I$ is the identity matrix. The total attention flow is obtained by multiplying the matrices between layers. Given that the layers in ViT are connected by using residual connections, the identity matrix $I$ is added to the layer Attention matrices. In multiple attention heads, three solutions are used to calculate the weights that affect them. The Attention Rollout suggests taking the average of the heads and discarding a certain percentage of weights by removing noise according to the weights' common denominator as the *Mean* fusion. The minimum or maximum weights (*Min* or *Max* fusion) are also proposed to determine the weights of the Attention layers.

## Reference

S1. Fawcett, T. An introduction to ROC analysis. **2006**, *27*, 861–874.
